# Evaluation of the abscopal-like effect of radioimmunotherapy on sentinel lymph nodes in H22 hepatocellular carcinoma via dynamic lymphography

**DOI:** 10.1186/s12885-026-16140-w

**Published:** 2026-05-07

**Authors:** Hui Wang, Dengyun Chen, Zhehan Liu, Xuefeng Zhao, Jinzhou Zhang, Dan Zhang

**Affiliations:** 1 Department of Nuclear Medicine, the First Affiliated Hospital of Anhui Medical University, No.218 Jixi Road, Hefei City, Anhui Province 230001 China; 2 First School of Clinical Medicine, Anhui Medical University, Hefei, 230032 China

**Keywords:** Lymphography, Abscopal effect, Irradiation, PD-1, Hepatocellular carcinoma

## Abstract

**Background:**

The synergistic suppressive effects of the radioimmunotherapy-induced abscopal effect on distant non-irradiated tumors have recently emerged as a research hotspot. However, studies on its specific regulatory role in adjacent sentinel lymph nodes (SLNs) remain limited. The deep location and small size of SLNs hinder the monitoring of treatment-related changes, underscoring the need for lymphography-based approaches. In this study, we aimed to evaluate the abscopal-like effect of radioimmunotherapy on SLNs in an H22 hepatocellular carcinoma mouse model via dynamic lymphography.

**Methods:**

H22 murine hepatocarcinoma cells were transplanted into the right hind foot sole of C57BL/6 mice. Fourteen days post-inoculation, mice with SLNs were selected and divided into six groups: control, isotype control Ig, anti-PD-1 alone, irradiation (IR) alone, irradiation + isotype control Ig, and IR + anti-PD-1. The volume and weights of tumors and non-irradiated SLNs were measured. The SLNs were imaged with ¹⁸F-fluorodeoxyglucose (^18^F-FDG) dynamic lymphography at 14, 21, and 28 days post-inoculation. CD8 immunohistochemistry assays were performed to assess CD8^+^ T cell infiltration.

**Results:**

In this mouse model, the IR + anti-PD-1 combination treatment exerted a marked suppressive effect on the growth of both irradiated tumors and non-irradiated SLNs, demonstrating a robust enhancement of the abscopal-like effect compared with the monotherapy groups. Mechanistic investigations demonstrated elevated CD8⁺ T cell infiltration in non-irradiated SLNs, suggesting that enhanced systemic anti-tumor immunity mediated the effect. Dynamic ¹⁸F-FDG PET lymphography enabled clear visualization of SLNs as early as 30 s post-injection, with sustained imaging clarity for over 30 min. This method also demonstrated decreased radioactive accumulation in irradiated tumors and non-irradiated SLNs, confirming an enhanced abscopal-like effect with the IR and anti-PD-1 combination approach.

**Conclusions:**

Our data demonstrate that the regression of SLNs adjacent to the irradiation field, mediated by the abscopal-like effect of radioimmunotherapy, can be sensitively and effectively tracked via dynamic ^18^F-FDG lymphography. Furthermore, our findings provide an effective and straightforward lymphographic approach with substantial translational potential for assessing the efficacy of the radioimmunotherapeutic abscopal-like effect against SLNs.

## Introduction

Metastasis is one of the most important factors contributing to poor prognosis in patients with hepatocellular carcinoma. As the “first station” of tumor lymphatic metastasis, the status of sentinel lymph nodes (SLNs), including their size, location, and activity, serves as a core basis for tumor staging, treatment strategy formulation, and prognosis evaluation. Programmed death-1 (PD-1) inhibitors, as representative drugs in the immunotherapy field, have become a key therapeutic approach for SLN-positive tumors, especially in neoadjuvant and adjuvant therapy settings [1–4]. Many basic science and clinical studies have confirmed that radiation therapy can recruit and accumulate effector T cells, reshape the tumor immune microenvironment, and support the transformation of “cold tumors” to “hot tumors”, thereby enhancing the efficacy of immunotherapy [5, 6].

In recent years, there has been interest in the abscopal effect of radiotherapy. This refers to the phenomenon where radiotherapy is administered to a single lesion in a cancer patient, but both the lesion within the irradiated field and non-irradiated distant lesions display regression, leading to effective disease control [7, 8]. The core mechanism of the abscopal effect, which has been experimentally demonstrated, is as follows: radiation induces primary tumor cells to release signals, such as tumor-associated antigens and damage-associated molecular patterns, which activate specific T cell immune responses. These activated T cells can then simultaneously attack the primary and metastatic lesions, leading to non-irradiated lesion shrinkage [9–10].

Although the synergistic effect of radiotherapy combined with immunotherapy has been confirmed, there is relatively little research regarding the specific regulatory effect of the radiotherapy-induced abscopal-like effect combined with immunotherapy on the SLNs adjacent to the primary tumor. SLNs are characterized by their deep location and small size, which render the observation of nodal changes during treatment challenging. This highlights the necessity of conducting lymphography-based studies.

As the most widely used clinical PET tracer, ¹⁸F-fluorodeoxyglucose (¹⁸F-FDG) is routinely administered intravenously for tumor metabolic imaging. Occasional subcutaneous extravasation during injection leads to radioactive accumulation in regional lymph nodes, implying its potential for lymphatic imaging. Direct evidence from one report [11] has demonstrated that intradermal tail injection of ¹⁸F-FDG in nude or C57BL/6 mice can clearly visualize inguinal lymph nodes within 5–10 min, with subsequent diffusion to mesenteric lymph nodes and systemic entry at 30–35 min post-injection.

In the present study, we aimed to establish a murine model of SLN metastasis, employ intradermal ¹⁸F-FDG injection-based PET lymphography for the dynamic monitoring of SLN alteration during concurrent irradiation and anti PD-1 treatment, and provide imaging evidence to optimize the combined therapeutic regimen for SLN-positive malignancies.

## Materials and methods

### Animals

Six-to-eight-week-old female C57BL/6 mice, weighing 19–21 g, were sourced from in-house breeding and maintained in the institution animal care facilities. Animal feeding and operations were conducted in accordance with the guidelines of the Institutional Animal Care and Use Committee (IACUC) of Anhui Medical University. The Animal Experimental Ethics Committee of Anhui Medical University approved the use of experimental mice in this study (Ethics Approval No.: LLSC20232073).

### Cell culture

H22 hepatocarcinoma cells were donated by Anhui Medical University. Cells were cultured in Dulbecco’s Modified Eagle’s Medium (DMEM; Gibco, Thermo Fisher Scientific, Waltham, MA, USA) supplemented with 10% fetal bovine serum (FBS; HyClone, Logan, UT, USA) and 1% penicillin-streptomycin (Invitrogen, Thermo Fisher Scientific). Cells were cultured at 37 °C in a humidified environment containing 95% air and 5% carbon dioxide.

### Reagents and equipment

The anti-PD-1 monoclonal antibody (clone 29 F.1A12) and isotype control Ig(InVivoMAb rat IgG2a isotype control) were obtained from BioXcell (Lebanon, NH, USA). The CD8 antibody (recombinant anti-CD8 alpha antibody) used in immunohistochemistry (IHC) experiments was purchased from Servicebio Technology Co., Ltd. (Wuhan, China) and used at a 1:200 dilution. Irradiation was performed using a VARIAN Vitallbeam medical linear accelerator (VARIAN Medical Systems Inc., Palo Alto, CA, USA) with a source-skin distance of 50 cm, irradiation field of 2 cm × 2 cm, and shielding of surrounding tissues. Imaging was performed using a Raycan TransPET Discoverist 180 micro PET (Raycan Technology Co., Ltd., Suzhou, China).

### Animal grouping and treatment

Tumor cells (1 × 10⁷ cells in 50 µL) were injected into the right hind foot sole of C57BL/6 mice. Two weeks later, mice with a spherical hard lump in their knee were selected. The SLNs were confirmed and visualized with ¹⁸F-FDG dynamic lymphography. The mice were randomly divided into six groups and treated as follows: (a) Control group (*n* = 6), treated with saline only; (b) Control Ig only group (*n* = 6), the isotype control Ig was administered as an intraperitoneal (i.p.) injection every two days over six days at a dose of 10 mg/kg; (c) Anti-PD-1 only group (*n* = 6), the anti-PD-1 monoclonal antibody was administered as an i.p. injection every two days over six days at a dose of 10 mg/kg; (d) Irradiation (IR) only group (*n* = 6), 24 Gy/3f, irradiation once every other day for six days with a fractional dose of 8 Gy. Only the primary tumors in the right hindlimb were irradiated, with the SLNs and other parts of the mice protected from radiation; (e) IR combined with control Ig group (*n* = 6), tumor-bearing mice were irradiated as described above and administered the isotype control Ig every other day for six days at a dose of 10 mg/kg; (f) IR combined with anti-PD-1 group (*n* = 6), tumor-bearing mice were irradiated as described above and administered the anti-PD-1 monoclonal antibody every other day for six days at a dose of 10 mg/kg. The tumor growth was monitored, with the primary tumor size measured with vernier calipers. Tumor volume (V) was calculated as follows: V (cm³) = length × width × width × 0.52.

### ¹⁸F-FDG PET dynamic lymphography and biodistribution studies

¹⁸F-FDG was supplied by Nanjing Andike Co., Ltd. (Nanjing, China). Approximately 3.7 MBq (10 µL) ¹⁸F-FDG was injected intradermally into and around the site of the foot tumors. Isoflurane (1% isoflurane-to-air mixture) was administered before the scan. A CT scan (1 mA, 50 kVP) was performed before injection for attenuation correction and anatomical localization. Dynamic PET imaging was acquired immediately after intradermal injection for as long as 30 min, with images reconstructed using the three-Dimensional Ordered Subsets Expectation Maximization Reconstruction (3D OSEM) combined with the Feldkamp-Davis-Kress (FDK) method and converted to units of percent injected dose per cubic centimeter (% ID/cc). The dynamic PET images were reconstructed with the following frame times: 5 s × 6, 10 s × 6, 30 s × 5, 60 s × 6, and 120 s × 10. Images were quantified by manually drawing regions of interest(ROIs) in the tumor and lymphnode areas. Lymphography was performed on days 14, 21, and 28 after tumor inoculation. The mice were humanely euthanized by cervical dislocation on day 28 after the last lymphography imaging was performed. The major organs, tumors, and SLNs were excised, washed, and wicked of excess water. Background-corrected counts per minute (CPM) of the excised tissues were measured using an automated Wizard γ-counter (Revvity, Waltham, MA, USA), decay-corrected to the time of radiotracer injection, and finally expressed as the percentage of the injected dose (% ID), as well as the % ID normalized to the tissue mass (% ID/g).

### IHC

After the mice were imaged and sacrificed on day 28, the tumors and SLNs were harvested, weighed, and fixed in 4% paraformaldehyde. Next, 4-µm-thick sections were cut from the samples, mounted on glass slides, and subjected to hematoxylin and eosin (H&E) staining. CD8 IHC staining (1:200 dilution) was performed on additional sections, with 3,3’-diaminobenzidine (DAB) as the chromogen. The percentage of positively stained cells relative to the total number of nucleated cells was calculated under high-power microscopic fields.

### Statistical analysis

Statistical analysis was performed using SPSS 16.0 software (SPSS Inc., Chicago, IL, USA). All normally distributed measurement data are presented as the mean ± standard deviation (SD). If most samples were normally distributed, then comparisons among multiple groups were conducted using one-way or repeated-measures analysis of variance (ANOVA), with pairwise comparisons between groups performed using the LSD-t test. For non-normally distributed data, non-parametric alternatives, including the Mann–Whitney U test (for two independent groups) and Kruskal–Wallis H test (for multiple independent groups), were applied. The association between in vivo PET-derived values and ex vivo biodistribution data was carried out with the Pearson correlation coefficient and linear regression. A *P*-value < 0.05 was considered statistically significant.

## Results

### H22 hepatocarcinoma cells serve as an ideal preclinical model for SLN metastasis research

The results demonstrated that H22 hepatocarcinoma cells yielded a markedly high SLN positivity rate, which reached 100% for animals inoculated with a concentration of 1 × 10^7^cells/50 µL. Notably, SLNs were successfully detected in all mice at 14 days post-tumor cell inoculation (Table 1).

**Table 1:** Sentinel lymph node (SLN) positivity rates at different inoculation concentrations

Tumor cell lines/Time	14 d	28 d
1 × 10^6^ H22, 50µL	3/6 (50% )	5/6 (83.3%)
1 × 10^7^ H22, 50µL	6/6 (100%)	6/6 (100%)

### Irradiation of the primary tumor combined with anti-PD-1 immunotherapy enhances the abscopal-like effect on SLNs in the H22 hepatocarcinoma mouse model

**Fig. 1:**
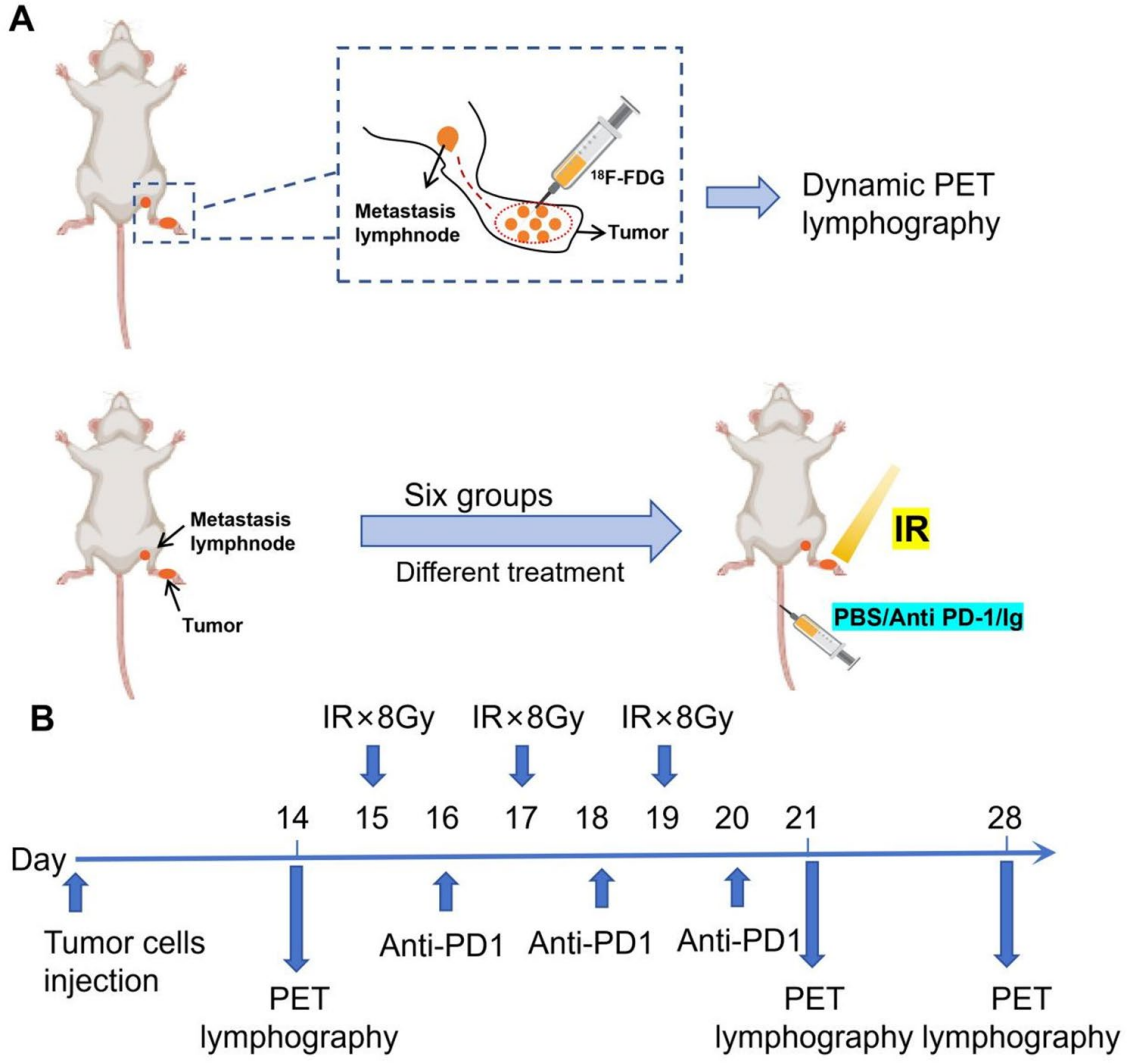
Schematic diagram of research methods. **A** A schematic showing the design of our animal experiment. H22 hepatocarcinoma cells were transplanted into the hind foot sole of C57BL/6 mice. Fourteen days post-inoculation, mice with sentinel lymph nodes (SLNs) were selected and divided into six groups for different treatments. The SLNs were imaged with ¹⁸F-fluorodeoxyglucose (^18^F-FDG) dynamic lymphography. **B** Timeline of the tumor implantation, treatment, and imaging schedule

**Fig. 2:**
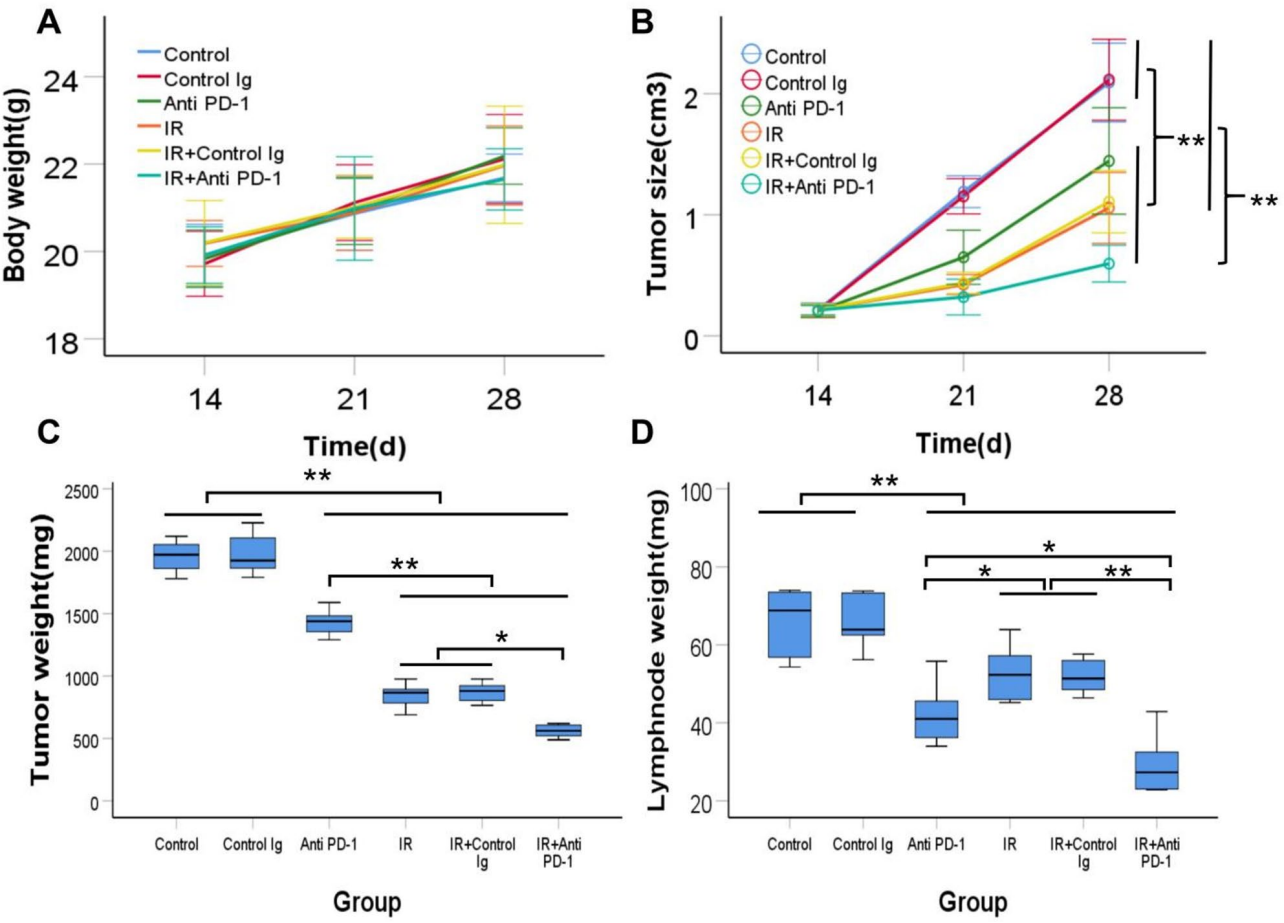
The irradiation combination with anti-PD-1 (IR + anti-PD-1) treatment enhances the abscopal-like effect in the H22 hepatocarcinoma mouse model. **A** Body weight curves (*n* = 6) showing no significant weight loss in the mice, even in the combination group. **B** Tumor growth curves. **C**, **D** Tumor weights at the study endpoint (day 28) (**C**) and sentinel lymph node (SLN) weights (**D**) (*n* = 6). The IR + anti-PD-1 group showed significant inhibition of irradiated tumors and non-irradiated SLNs compared with the control and monotherapy groups. *P < 0.05, **P < 0.01

H22 tumor-bearing mice with confirmed SLN metastasis were divided into six groups and administered different treatment regimens starting on day 14 post-tumor cell inoculation. The treatment protocol is outlined in Fig. 1. No significant differences in body weight were observed among the tumor-bearing mice across all groups throughout the treatment period, mitigating any potential concern regarding adverse effects of this combination therapy (Fig. 2A).

Notably, on day 21 (post-treatment completion), the primary tumor volume growth rates in the four treatment groups were significantly slower than those in the control groups(*P* < 0.01), while the primary tumor volume in the isotype Ig control group was comparable to that in the blank control group. However, the differences between the IR and anti-PD-1 monotherapy groups and the combination treatment group on day 21 were not significant. The inhibitory effect of the combined therapy was further amplified on day 28, with the primary tumor volume and weight in the irradiation combined with anti-PD-1 group being significantly lower than those in all other groups (*P* < 0.01). The primary tumor volumes and weights from the control, control Ig, anti-PD-1, IR, IR + control Ig, and IR + anti-PD-1 groups were as follows: 2.09 ± 0.31 cm^3^, 1.97 ± 0.12 g; 2.12 ± 0.32 cm^3^, 1.97 ± 0.16 g; 1.44 ± 0.42 cm^3^, 1.43 ± 0.11 g; 1.06 ± 0.28 cm^3^, 0.85 ± 0.10 g; 1.08 ± 0.23 cm^3^, 0.87 ± 0.08 g; 0.60 ± 0.14 cm^3^, 0.56 ± 0.05 g, respectively (Table 2**/**3, Fig. 2B**/**C).

**Table 2:** Tumor volumes in the six groups at three time points

Group/Time	Control	Control Ig	Anti PD−1	IR	IR+Control Ig	IR+Anti PD−1	F	*P*-value
Day14	0.23 ± 0.06	0.21 ± 0.05	0.21 ± 0.05	0.21 ± 0.05	0.22 ± 0.05	0.21 ± 0.04	0.04	0.99
Day21	1.19 ± 0.13	1.19 ± 0.11	0.65 ± 0.21	0.42 ± 0.08	0.44 ± 0.08	0.32 ± 0.14	52.46	0.00^**^
Day28	2.09 ± 0.31	2.12 ± 0.32	1.44 ± 0.42	1.06 ± 0.28	1.08 ± 0.23	0.60 ± 0.14	25.59	0.00^**^

**P < 0.01

**Table 3:** Endpoint tumors and the sentinel lymph nodes weight in the six groups

Group/Weight	Control	Control Ig	Anti PD−1	IR	IR+Control Ig	IR+anti PD−1	F	*p*
Tumor(g)	1.97 ± 0.12	1.97 ± 0.16	1.43 ± 0.11	0.85 ± 0.10	0.87 ± 0.08	0.56 ± 0.05	184.29	0.00^**^
Lymph node(mg)	66.03 ± 8.79	65.60 ± 6.82	42.27 ± 8.22	52.82 ± 7.52	51.87 ± 4.62	29.30 ± 7.82	21.58	0.00^**^

**P < 0.01

Because of the deep location of SLNs, accurately measuring the volume was difficult. After imaging on day 28, tumors and SLNs were harvested and weighed. The SLN weights from the control, control Ig, anti-PD-1, IR, IR + control Ig, and IR + anti-PD-1 groups were as follows: 66.03 ± 8.79 mg, 65.60 ± 6.82 mg, 42.27 ± 8.22 mg, 52.82 ± 7.52 mg, 51.87 ± 4.62 mg, 29.30 ± 7.82 mg, respectively (Table 3). Intergroup comparisons showed that the anti-PD-1 monotherapy, IR monotherapy, and combined treatments all exerted inhibitory effects on both the tumors and SLNs compared with the control groups (*P* < 0.01), with the IR combined with anti-PD-1 treatment significantly inhibiting SLN growth compared with the IR and anti-PD-1 monotherapy groups (*P* < 0.05) (Fig. 2D).

### Dynamic ¹⁸F-FDG PET lymphography confirms an enhanced abscopal-like effect by IR combined with anti-PD-1 immunotherapy in the H22 hepatocarcinoma mouse model

**Fig. 3:**
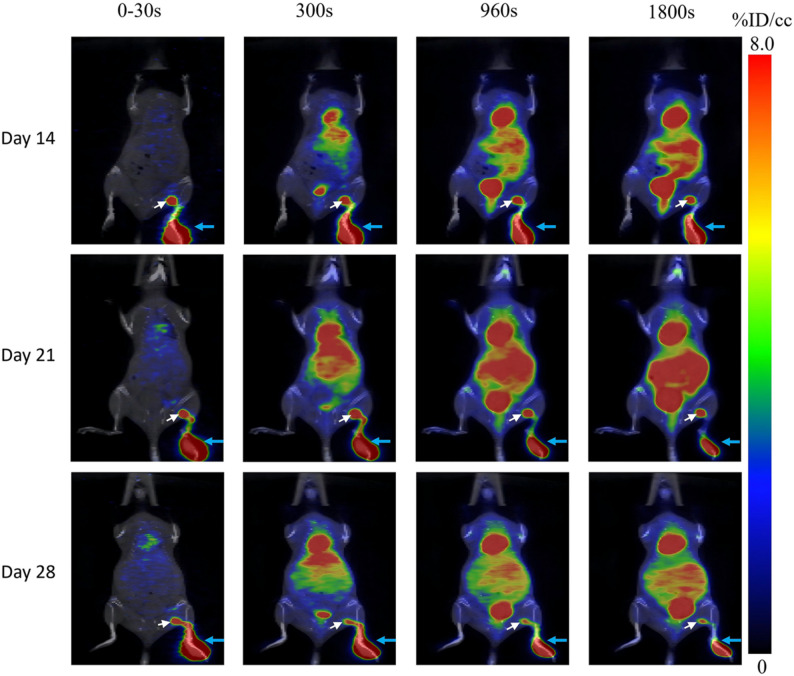
Dynamic ¹⁸F-fluorodeoxyglucose (^18^F-FDG) lymphography images from the IR + anti-PD-1 group. Images at three timepoints (14, 21, and 28 days post-tumor cell inoculation) showing reduced radioactive accumulation in irradiated tumors (blue arrows) and non-irradiated sentinel lymph nodes (SLNs; white arrows). The SLNs can be clearly visualized as early as 30 s post-injection, with sustained imaging clarity for over 30 min

**Fig. 4:**
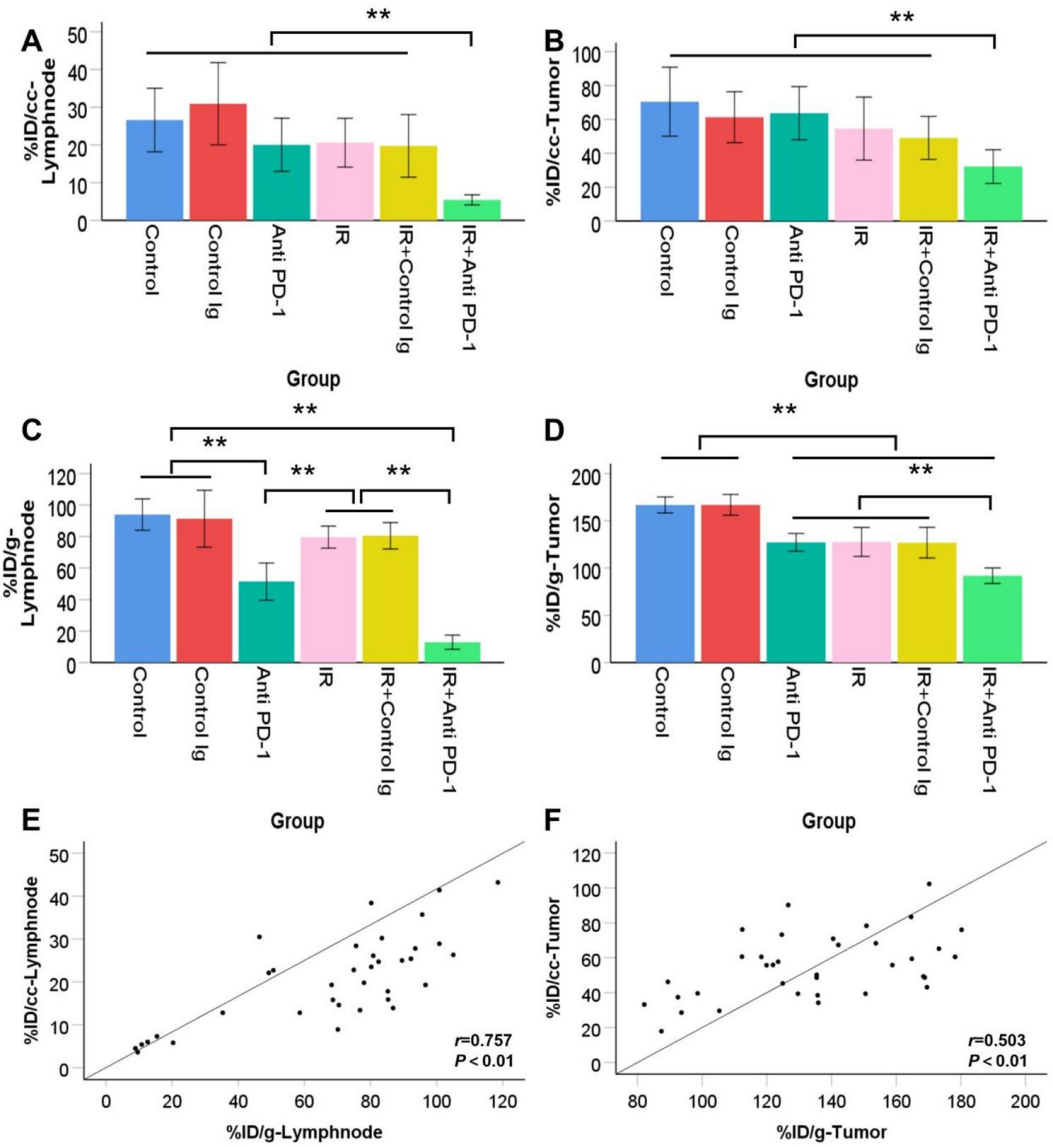
Quantitative analysis of ^18^F-FDG uptake in lymphnodes and tumors across different groups after intradermally administration. **A**-**D** % ID/cc and % ID/g uptake in lymphnodes and tumors across Control, Control Ig, Anti-PD-1, IR, IR+Control Ig, and IR+Anti PD-1 groups. **E**-**F** Correlation between % ID/cc and % ID/g uptake in lymph nodes (*r* = 0.757, *P* < 0.01) and tumors (*r* = 0.503, *P* < 0.01). The IR + anti-PD-1 combination group showed lower % ID/g in the irradiated tumors and non-irradiated SLNs compared with the control and monotherapy groups, with a similar trend observed for % ID/cc. **P < 0.01

In the present study, SLNs were rapidly and distinctly visualized via dynamic PET imaging at 30 s following intradermal injection of ¹⁸F-FDG into and around the foot tumor sites. Subsequently, the imaging agent gradually entered the blood, then the radioactivity in the heart, liver, spleen, abdominal intestines, and urine in the bladder gradually increased. At 30 min, significant radioactivity retention was observed in the SLNs with a high target-to-background ratio. Over time, the radioactivity at the dorsal foot tumor injection site faded. Dynamic PET lymphography could sensitively track changes in the SLNs at different time points (days 14, 21, and 28) (Fig. 3). After normalization to tissue mass for the calculation of the % ID/cc values, the high lymph node-to-other tissue uptake ratios were consistent with the clear imaging delineation of the SLNs. Specifically, the endpoint IR + anti-PD-1 combination group showed lower % ID/g and % ID/cc in irradiated tumors and non-irradiated SLNs compared with the control and monotherapy groups (*P* < 0.01) (Fig. 4A-D). Moreover, statistical analysis demonstrated a close correlation between the PET parameter % ID/cc and the ex vivo biodistribution data % ID/g, further supporting the feasibility and effectiveness of dynamic ¹⁸F-FDG PET lymphography for evaluating the efficacy of the radioimmunotherapeutic abscopal-like effect against SLNs (Fig. 4E**/F**).

### The IR and anti-PD-1 combination slightly increases CD8^**+**^ T cell infiltration in irradiated H22 tumors and non-irradiated SLNs

**Fig. 5:**
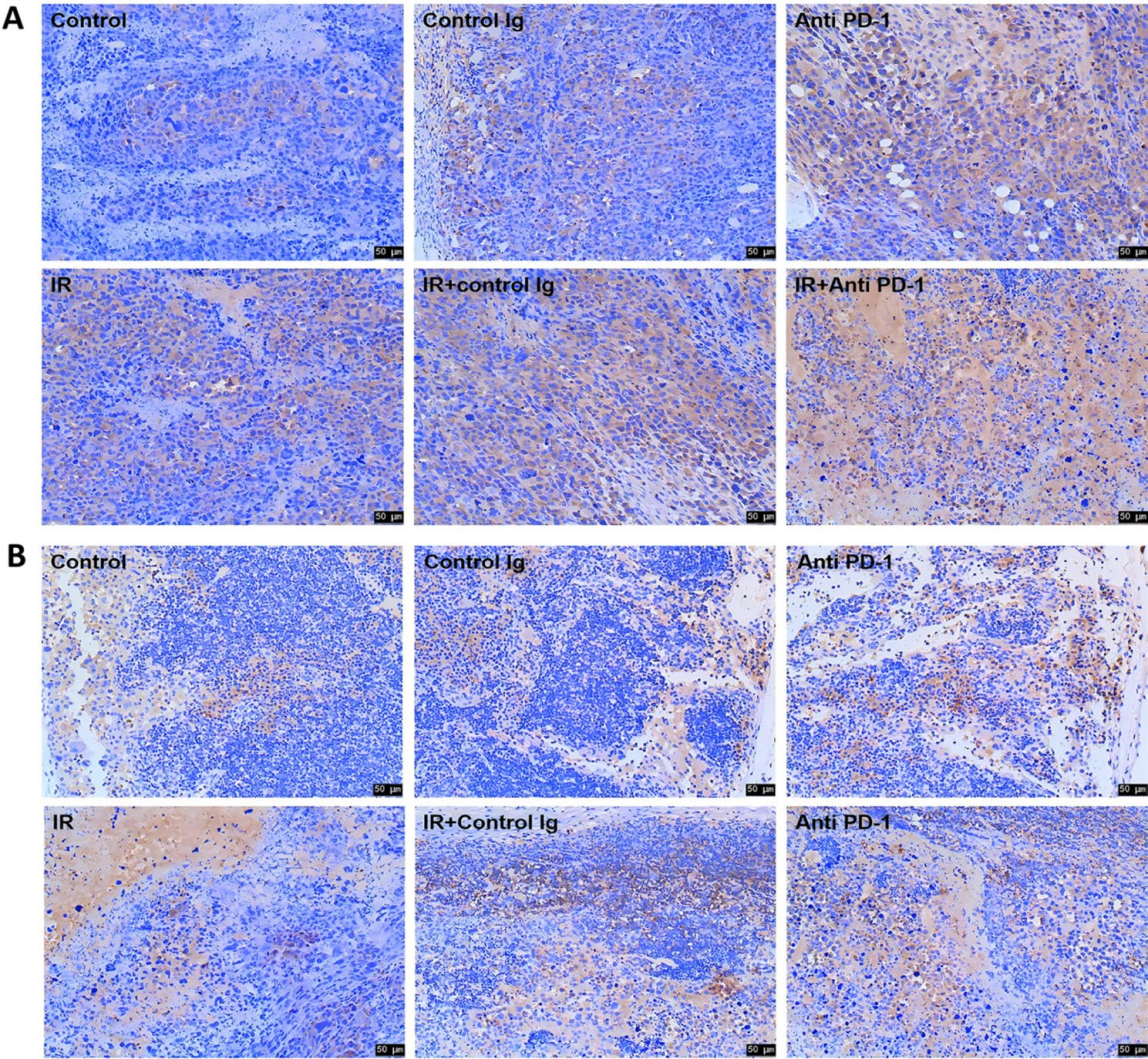
Representative CD8 immunohistochemistry (IHC) images. Images are shown at 200⋅ magnification. CD8 IHC images of irradiated H22 tumors (**A**) and sentinel lymph nodes (**B**)

**Fig. 6:**
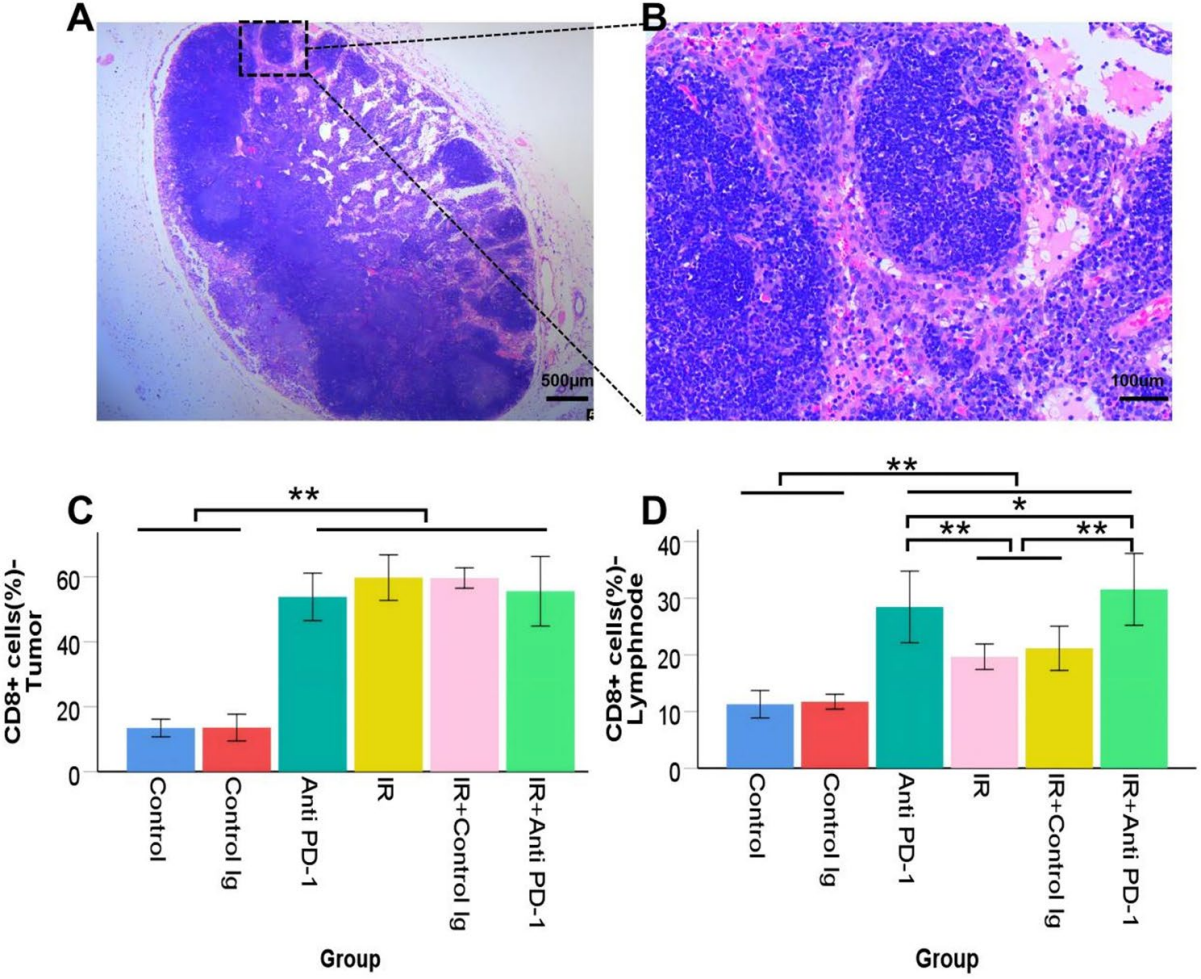
Representative hematoxylin and eosin (H&E) staining results and CD8 immunohistochemistry (IHC) quantification. H&E images of sentinel lymph nodes (SLNs; **A**,**B**) and quantification of CD8-positive (%) tumors (**C**) and SLNs (**D**) by IHC analysis. *P < 0.05, **P < 0.01

The IHC results showed that CD8⁺ cells in the primary tumors were mainly concentrated in the tumor parenchyma, while CD8⁺ cells in the SLNs were mainly distributed in the paracortical area (Fig. 5A/B). HE staining confirmed tumor cell infiltration in SLNs (Fig. 6A**/**B). Anti-PD-1 monotherapy, IR monotherapy, and IR combined with anti-PD-1 could increase the number the CD8⁺ cells in the tumors and SLNs (Fig. 6C/D). Although the differences in the irradiated primary tumors were not statistically significant among the treatment groups, the IR combined with anti-PD-1 group exhibited a significantly higher level of CD8⁺ T cell infiltration in non-irradiated SLNs compared with the control and monotherapy groups (*P* < 0.05).

## Discussion

In the present study, we evaluated the radioimmunotherapy-induced abscopal-like effect on adjacent SLNs in an H22 hepatocellular carcinoma mouse model using dynamic ^18^F-FDG lymphography. Irradiation was administered to primary foot tumors in H22 model mice, either alone or in combination with anti-PD-1 immunotherapy. We then observed and calculated changes in the volume and weight of primary tumors and SLNs. The results showed that IR combined with anti-PD-1 treatment not only exerted a stronger inhibitory effect on the irradiated primary tumors, but also resulted in a lower weight of non-irradiated SLNs compared with the other treatment groups. Other researchers [12–14] have also confirmed that irradiation combined with immunotherapy can promote tumor cell death and improve therapeutic effects. Yoo et al. [15] demonstrated that administering 16 Gy in two fractions could more effectively inhibit the growth of both irradiated and non-irradiated tumors with higher cytotoxic T cell infiltration than 8 Gy did in a single fraction. In the present study, irradiation was administered in a fractional manner: once every other day for six days, with a fractional dose of 8 Gy. Additionally, only the primary tumors in the right hindlimb were irradiated, with the SLNs and other parts of the mice protected from the radiation. IHC assays confirmed enhanced CD8^+^ T cell infiltration in the irradiated tumors and non-irradiated SLNs. In the irradiated primary tumors, all active treatment groups (Anti-PD-1, IR, IR + Control Ig, and IR + Anti-PD-1) exhibited markedly higher CD8⁺ T cell proportions than the Control and Control Ig groups. This confirms that both anti-PD-1 treatment and local irradiation can independently enhance tumor-localized anti-tumor immunity, consistent with the known abilities of irradiation to trigger immunogenic cell death and anti-PD-1 to reverse T cell exhaustion in the tumor microenvironment [16, 17]. Notably, the IR + Anti-PD-1 group maintained robust CD8⁺ infiltration, indicating that the combination does not impair local anti-tumor T cell responses.

The non-irradiated SLN results are critical for demonstrating the abscopal-like effect in this model. While the monotherapies (Anti-PD-1, IR) and IR + Control Ig increased the number of SLN CD8⁺ cells relative to the controls, the IR + Anti-PD-1 group showed the most prominent degree of CD8⁺ infiltration in non-irradiated SLNs. Notably, this level was significantly higher than that of the IR + Control Ig group. This observation directly supports the notion that combining local IR with anti-PD-1 therapy can potentiate the abscopal-like effect: local irradiation releases tumor antigens to prime T cells, while PD-1 inhibition prevents the exhaustion of these activated T cells, enabling their trafficking to adjacent, non-irradiated SLNs. Mechanistic investigations showed increased CD8⁺ T cell infiltration in non-irradiated SLNs. Although these results imply enhanced systemic antitumor immune responses, the functional status of the infiltrating T cells was not directly assessed in the present study. Multiple studies [18–22] have shown that radiotherapy can activate immature T cells to differentiate into CD8^+^ effector T cells in both primary tumors and abscopal lesions, even leading to the reversal of tumor immune desertification and immunotherapy resistance. Thus, the generation of anti-tumor effector CD8^+^ T cells appears to be a key mechanism underlying the abscopal effect.

In the present study, ¹⁸F-FDG PET dynamic lymphography could clearly visualize SLNs as early as 30 s after injection, with a high target-to-background ratio and clear images. ¹⁸F-FDG PET lymphography enables rapid imaging with clear images and maintains good retention at 30 min, providing an ideal imaging time window. The mean weight of the SLNs harvested from the IR + anti-PD-1 group on day 28 was only 29.3 mg, with a diameter of approximately 5 mm. Despite this small size, these SLNs could still be clearly visualized, indicating the high sensitivity of ¹⁸F-FDG PET lymphography. The radioactivity at the dorsal foot injection site faded significantly at 30 min post-injection, while radioactivity gradually accumulated in the heart, liver, spleen, and bladder. This suggests that the imaging agent diffused rapidly, entered the blood gradually, and was excreted through the urinary system. Mueller and colleagues [23] pioneered positron lymphography, in which ¹⁸F-FDG was injected interstitially into the uterine cervix of 23 patients with uterine or cervical cancer on the day of surgery. Its rapid transport through the lymphatic vessels to the SLNs was then visualized with dynamic PET/CT. Collectively, the current and previous data demonstrate that FDG lymphography represents an effective tool for diagnosing metastatic lymph nodes.

Despite the advantages of this imaging method, it suffers from a critical drawback: quantitative evaluation of SLNs remains challenging. The semi-quantitative index uses the standardized uptake value(SUV), which is used in conventional PET imaging to normalize the radioactivity concentration in target tissues to the injected dose of the radiotracer, while accounting for patient body weight to eliminate individual differences [24–28]. This value is calculated following intravenous tracer injection and is based on the equilibrium distribution of ¹⁸F-FDG between plasma and tissues. In the present study, PET images were reconstructed and converted to units of % ID/cc. After the final imaging session, we calculated background-corrected CPM, which were decay-corrected to the time of radiotracer injection. Ultimately, these values were expressed as the percentage of the injected dose normalized to tissue mass (% ID/g). At the experimental endpoint, the IR + anti-PD-1 group exhibited lower % ID/cc and % ID/g values in both irradiated tumors and non-irradiated SLNs compared with the control and monotherapy groups. Statistical analysis demonstrated a close correlation between the PET parameter % ID/cc and the ex vivo biodistribution data % ID/g, further supporting the feasibility and effectiveness of dynamic ¹⁸F-FDG PET lymphography for monitoring the therapeutic response of SLNs.

Several limitations of this study should be acknowledged. First, H22 hepatoma is allogeneic in C57BL/6 mice, which may exaggerate immune responses. Second, although we used CD8 IHC analyses to demonstrate elevated CD8^+^ T cell infiltration in irradiated tumors and SLNs after therapy, lack of functional immune assay data, such as flow cytometry, remains a limitation. Third, further research is warranted to identify more optimal non-invasive quantitative parameters for lymphography.

## Conclusions

Overall, the abscopal effect represents potentially transformative implications for metastatic disease management. Our work in the current study demonstrated that the regression of SLNs beyond the irradiation field, driven by the radioimmunotherapy-derived abscopal-like effect, can be sensitively and effectively tracked through dynamic ^18^F-FDG lymphography. Our study provides an effective lymphography method for evaluating the efficacy of the radioimmunotherapeutic abscopal-like effect on SLNs.

## Data Availability

The datasets used and/or analyzed during the current study are available from the corresponding author on reasonable request.

## Abbreviations

^18^F-FDG: ^18^F-Fluorodeoxyglucose
PET/CT: Positron emission tomography/computed tomography
IR: Irradiation
PD-1: Programmed death-1
CD8: Cluster of differentiation 8
RPMI: Roswell Park Memorial Institute
SLNs: Sentinel lymph nodes
TAAs: Tumor-associated antigens
DAMPs: Damage-associated molecular patterns
IACUC: Institutional Animal Care and Use Committee
%ID: Percentage of the injected dose
%ID/g: Percent injected dose per gram of tissue
%ID/cc: Percent injected dose per cubic centimeter
CPM: Counts per minute
SUV: Standardized uptake value
Gy: Gray
IHC: Immunohistochemistry
H&E: Hematoxylin and eosin
DAB: Diaminobenzidine
ANOVA: Analysis of variance
